# Simple Screening of *Listeria monocytogenes* Based on a Fluorescence Assay via a Laminated Lab-On-Paper Chip

**DOI:** 10.3390/bios7040056

**Published:** 2017-11-28

**Authors:** Kankanit Pisamayarom, Annop Suriyasomboon, Piyasak Chaumpluk

**Affiliations:** 1 Laboratory of Plant Transgenic Technology and Biosensor, Department of Botany, Faculty of Science, Chulalongkorn University, Bangkok 10330, Thailand; toomkankanit@gmail.com; 2 Program in Biotechnology, Faculty of Science, Chulalongkorn University, Bangkok 10330, Thailand; 3 Department of Animal Husbandry, Faculty of Veterinary Science, Chulalongkorn University, Bangkok 10330, Thailand; annop.suriyasomboon@gmail.com

**Keywords:** Lab-on-paper chip, Listeria monocytogenes, Detection, LAMP, *hly* gene, frozen seafood

## Abstract

Monitoring food safety is essential for protecting the health and safety of consumers. Conventional methods used are time consuming and laborious, requiring anywhere from three to seven days to obtain results. Thus, better monitoring methods are required. In this study, a laminated lab-on-paper chip was developed, and its use for the screening of ready-to-eat seafood was demonstrated. The assay on a chip was based on loop-mediated isothermal DNA amplification (LAMP) of the *hly* gene of *Listeria monocytogenes* and fluorescence signal detection via SYBR Gold^TM^. Overall assay processes were completed in 4.5 h., (including 3.5 h. incubation for the bacteria enrichment, direct DNA amplification with no DNA extraction, and signal detection), without relying on standard laboratory facilities. Only positive samples induced fluorescence signals on chip upon illumination with UV light (λ = 460). The method has a limit of detection of 100 copies of *L. monocytogenes* DNA per 50 g of sample. No cross-reactivity was observed in samples contaminated with other bacteria. On-site monitoring of the seafood products using this chip revealed that one of 30 products from low sanitation vendors (3.33%) were contaminated, and these agreed with the results of PCR. The results demonstrated a benefit of this chip assay for practical on-site monitoring.

## 1. Introduction

Several changes in food processing, food storage and patterns of consumption have increased the rates and risks of food poisoning. This increased risk has manifested as an increasing incidence of illnesses arising from foodborne pathogens worldwide [1]. In 2010, the WHO had released initial findings, showing hundreds of millions of people worldwide were getting sick from contaminated food [2]. At the industry level, self-regulation in risk analysis is required during food production [3,4,5]. One measure is the introduction of the Hazard Analysis and Critical Control Point (HACCP) systems, requiring management of microbial issues through the identification and monitoring of hazards by the food industry. To implement this measure, a system for regular monitoring of food at all levels is required.

*Listeria monocytogenes* is a foodborne bacterium that causes listeriosis. It has been found in both raw and processed food samples, including dairy products, meats, vegetables and seafood [6,7]. Infection with this bacterium often produces non-specific initial manifestations; without prompt antibiotic therapy, it can have severe clinical consequences, occasionally including death [8,9]. The classical assay for *L. monocytogenes* utilizes a standard culture method and bacteriological tests via colony isolation on selective agar and further biochemical and serological confirmation, which takes three to five days to obtain a result. Alternatively, immunoassays and polymerase chain reaction (PCR) methods have been incorporated to accelerate detection. Although PCR-based methods are the most widely used, and several PCR systems are available [10,11,12], this approach requires a thermo cycler to separate double-stranded DNA and amplify nucleic acids and is, therefore, not suitable for on-site monitoring as needed for safety control programs [13].

In developing countries, a solution for food testing with high specificity, sensitivity, and reliability at an affordable cost is urgently needed, especially for users without reliable access to expertise and laboratory facilities. To this end, an approach based on a point-of-care test (POCT) for working in resource limited settings is desirable. Thus far, several platforms, including immunological, molecular and biological tests using microfluidic and paper-based devices (μPAD), are available [14]. The latter have attracted particular interest due to the original cellulose properties in paper, which allow the chip structure to be easily fabricated, compatible with the majority of biological samples, lightweight and easy to use, all allowing a new era of highly-tailorable chips at a low cost [15,16].

Several approaches have been employed to fabricate paper-based chips, starting with the pioneering work of Whitesides, who implemented photoresistant SU-8 together with photolithographic techniques [17,18]. Then, the technique moved to a plotting method, using inkjet or toner printing, cutting, and wax patterning [19,20,21]. Although these methods enabled the customization of chips for specific purposes, most of them still required specific tools and reagents that are not suitable for resource- and experience-limited settings.

Plastic lamination was first introduced to tailor the μ-PAD [22]. It involved only common stationery, a cutter, and a lamination process. Simple lamination enabled the chip to be made easily as compared to that of a typical ID card preparation and provided advantages in terms of the low initial investment and operation cost and the ability to fabricate chips in a resource-limited setting with little to no professional skills required.

In this study, we successfully designed a laminated chip suitable for Listeria nucleic acid detection with on-chip DNA amplification and signal detection performed in sequence. In the classical method, both differ drastically in terms of the buffer actions, temperatures and reaction conditions. Previously, loop-mediated isothermal amplification (LAMP) of DNA [23] was applied for nucleic acid amplification on the chip instead of PCR, and the DNA signals were later detected via a colorimetric assay [24,25]. Although a colorimetric assay reported recently was based primarily on gold nanoparticles (AuNPs), it was not suitable for paper-based chip platforms due to several particle stability problems and incompatibility with the cellulose matrix of paper [16].

This study reports the first attempt at the development of an on-chip assay combining DNA amplification via LAMP and fluorescence detection via a DNA binder into a simple paper-based lab-on-a-chip platform fabricated using fishing line and Whatman^TM^ filter paper via simple plastic lamination. The application of this chip for on-site detection of Listeria in seafood and ready-to-eat seafood specimens was also demonstrated.

## 2. Materials and Methods

### 2.1. Chip Design and Fabrication

**Figure 1:**
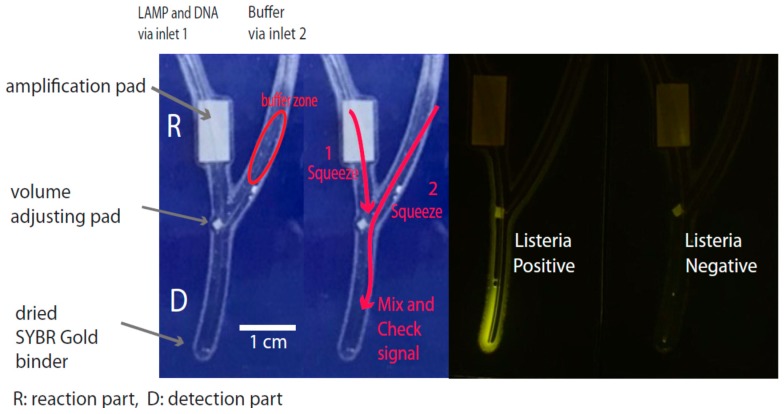
Design of the chip and its structure. The chip was divided into two parts, one for DNA amplification (**R**) and the other for detection (**D**). Both were connected with a channel made of fishing line. The LAMP reagent and DNA sample were introduced via inlet 1, and the blue AgNPLs/probe was introduced via inlet 2. DNA amplification occurred after sealing the inlets with PE stickers and incubating at 63 °C for 30 min. For DNA detection, the amplified products were squeezed to touch a volume adjusting pad first (**arrow 1**); later, the buffer solution was squeezed through to collect the DNA from the volume adjusting pad (**arrow 2**), spun down, mixed, and placed on a UV light source. The fluorescence in a positive sample could be visualized by the naked eye.

In LAMP, DNA amplification is carried out at pH 8.8 with KCl and MgSO_4_ in solution, and the fluorescence DNA signal detection step also requires a diluted DNA product at a fixed DNA binder concentration. Thus, the design of the chip for the DNA assays required two separate compartments that look like a Y character, one for the amplification and the other for signal detection (Figure 1). The chip had dimensions of 2.6 × 4.75 cm^2^. The reaction part (R) comprised a single reaction zone with a rectangular shaped paper pad (~0.7 × 0.5 cm^2^) for nucleic acid amplification, with a connection channel made from fishing line (No 28; 0.28 mm in diameter) to deliver the amplified DNA solution. Paper was used to accommodate a problem of undesirable flowing of reagent in the chip structure during preparation and use, and to control the volume post-amplification. There was a small square paper near inlet 1 and a volume adjusting pad to control the delivery of DNA products at approximately 1–1.5 µL. The detection part (D) was also made by fishing line, where the Y bottom contained a SYBR Gold^TM^ DNA binder, and the right arm of Y contained buffer. The DNA binder reacted to the LAMP DNA products, providing fluorescence signals after exposure to UV light. All the paper pads were made of a layer of Whatman^TM^ filter paper No. 1. Fabrication of the chip was carried out on a clean bench. All materials, except the laminated film and plastics, were sterilized at 121 °C and 15 psi for 20 min to reduce nuclease contamination. Plastics and fishing line were surface sterilized with 1% sodium hypochlorite for 20 min and washed using sterile deionized water and dried on a clean bench. New laminated diethyl pyrocarbonate (DEPC)-treated film was used to ensure nuclease-free conditions. For signal detection, the bottom of the Y was initially filled with 2 µL of 1000× diluted SYBR Gold^TM^ and then dried at room temperature before setting. Complete fabrication was performed by plastic lamination at 130 °C using a 125-mm pouch film and a pouch laminator (LMR-220) (Lamirel, Moscow, Russia). The inlet lines were made by placing a fishing line into the structure and then sealing with lamination. The closing of each inlet was performed using a polyethylene (PE) sticker.

### 2.2. Establishment of Nucleic Acid Amplification

Several genes related to pathogenicity were reviewed, and candidate genes were selected based on sequence similarity among the strains *(Listeria)* and the distance among the non-*Listeria* groups [26]. Briefly, sequences were retrieved from GenBank accession number AF253320.1. The sequences were then aligned using the ClustalW alignment program [27]. Pathogenicity criteria were reviewed from previously-published data [28]. The data on the risk assessment determined the levels of *L. monocytogenes* in food and ready-to-eat foods from several countries. A candidate gene was selected based on the Tm, E value, and nucleotide sequence. Once selected, specific primers for LAMP were designed based on the program Primer Explorer Version 4 (Fujitsu, Tokyo, Japan). The specificity of each primer and the detailed melting temperature were screened using the Basic Local Alignment Search Tool (BLASTn) [29] and oligocalculator [30]. Oligonucleotide probes specific to the target DNA were then designed based on the DNA sequence between the amplicon of LAMP. Details for the primers and probes are shown in Table 1. For a comparative study, the PCR method using primers specific to the *hly* gene (Table 1) was used as a reference, as described elsewhere [31].

Amplification of the target *hly* gene from the extracted DNA was performed with this primer set. The reaction mixture was comprised of 40 mM Tris pH 8.8, 20 mM KCl, 5 mM MgSO_4_, 0.2% Tween 20, 0.1% Triton X-100, 1.6 M Betaine, 5 mM dNTP, 8 U *Bst* DNA polymerase (New England Biolabs, MA, USA), 5 pmol each of F3 and B3 primer and 40 pmol each of FIP and BIP primer, as described [23]. After incubation at 63 °C for 30 min, the DNA products were examined by gel electrophoresis using 2.5% agarose in TAE buffer. For comparison with the PCR, one microliter of extracted DNA was amplified by PCR [32]. The DNA products were separated by electrophoresis on a 1.5% agarose gel in TAE buffer.

On-chip LAMP amplification was carried out by adding a 10 μL volume of the above LAMP reaction into the chip via inlet 1 (Figure 1), followed by sealing and incubating at 63 °C for 30 min in a water bath. Once finished, the results of the DNA products were examined as described in Section 2.4.

To construct the plasmid DNA for the reference positive control, the DNA products corresponding to the *hly* gene were cloned into a TA vector as described (Invitrogen, Madison, WI, USA). This vector was transformed into *Escherichia coli* [33], and the nucleotide sequence was determined. Known copy numbers of plasmid as a positive reference were prepared as described [34]. In the test, *L. monocytogenes* and non-*L*. *monocytogenes* DNA were amplified and compared with the PCR products, as described elsewhere [31]. The sensitivity and specificity of the LAMP technique were also evaluated based on the DNA copy number, as described [35], and by spiking live *L. monocytogenes* (10-fold dilutions from 10^7^ to 1 CFU) directly onto 50 g of frozen salmon, followed by detection after 3.5 h of enrichment.

### 2.3. Test Specimens

For LAMP amplification, 30 frozen seafood and ready-to-eat specimens were collected from modern-type supermarkets and local weekend markets in Bangkok, Thailand, focusing on tuna, salmon, and shrimp, and their products, which had been found to contain *Listeria* in the past. Strains of *L. monocytogenes*, as well as other non-*L. monocytogenes* strains, were collected from reliable sources and used throughout the study (Table 2). These strains included seven *L. monocytogenes* strains and other pathogenic strains of bacteria, including *L. innocua, L. ivanovii, and L. welshimeri*, *Vibrio cholera, V. parahaemolyticus, Salmonella enteritidis, E. coli* O157, Enterotoxigenic *E. coli* (ETEC), Enteropathogenic *E. coli* (EPEC)*, Pseudomonas putida*, and *Shigella flexneri*, from the Department of Medical Sciences, Ministry of Public Health Thailand (DMST) and Food Research and Testing Laboratory (FRTL), Faculty of Science, Chulalongkorn University, Thailand. For maintenance, the strain stocks were cultured separately in 3 mL of Luria Broth (LB) in 30 mL glass bottles and incubated at 37 °C for 16 h. For long-term maintenance, cultures were stored in 20% glycerol and kept at −20 °C.

For the bacterial strains, genomic DNA extraction was carried out on overnight cultures using standard phenol extraction [33]. The DNA was then treated with RNase A, purified on a Wizard^®^ column and used in experiments. For the bacteria from food samples, specimens were obtained by swabbing with a sterile cotton ball (2 cm in diameter) and enriching for 3.5 h at 37 °C in 2.5 mL of Terrific Broth [33]. The bacterial culture was then harvested in a microcentrifuge tube, and the sample was collected via centrifugation at 6000 rpm. Only 1 µL of the pellet was used as the specimen for direct on-chip amplification without DNA extraction.

### 2.4. Signal Detection via Fluorescence Assay

For standard DNA detection, the amplified DNA products from LAMP were separated via 1.5% agarose gel electrophoresis in TAE buffer and ethidium bromide staining. On-chip DNA detection was based on a fluorescence assay. In the SYBR Gold^TM^ test, a cyanine dye was used with 2 μL of a 10^−3^ concentration, as described in an earlier section. SYBR Gold^TM^ is the most sensitive fluorescent DNA binder. From the product datasheet, when it is desiccated and protected from light at –20 °C, it is stable more than several months. After on-chip nucleic acid amplification was completed, the amplification pad was pressed and squeezed down until the solution moved to the volume adjusting pad, which was designed to have a small absorption area of 1 × 1 mm^2^ for volume transferring. This made the absorbed solution approximately 1 µL compared to the volume adjusting pad. Next, the buffer solution from the right part was squeezed to pass through the volume adjusting pad down to the bottom of Y, where SYBR Gold^TM^ was previously dried. The buffer was used to control the pH to be between 7 and 8 to ensure the best condition for the SYBR Gold^TM^ reaction. This resulted in 1 µL of the DNA solution being transferred, diluted, and mixed into the SYBR Gold^TM^ solution (Figure 1). For signal detection, the solution was mixed by snapping the bottom part of the Y shape with a finger, and the entire chip was then placed on a UV LED light illumination box (λ = 460 nm) and checked for its fluorescence signal at room temperature.

### 2.5. Routine Operation of the Chip

Preparation of the ready-to-use chips for routine operation at on site was performed by adding 5 µL of 2× LAMP solution to the amplification pad via inlet No. 1, adding 0.1× phosphate buffer via inlet No. 2, sealing each of the inlets, and storing the chip at −20 °C in aluminum bags until use. If the chip was intended for use, activation of the chip was performed by removing a sticker, adding 5 µL of nucleic acid samples to the pad via inlet No. 1 using a crystal tip, and sealing both inlets with a plastic sticker or with a screw compressor clamp. Sealing this way would allow the chip unit to be closed tightly. The whole chip unit was then placed in a plastic bag and dipped in water in a closed polyurethane box at 63 °C for 30 min for reaction incubation. This would allow the DNA to be amplified. For fluorescence signal detection, the whole chip unit was removed, pressed to deliver the solution to the adjusting pad, mixed with buffer and DNA binder, and checked for its fluorescence signal as previously described.

### 2.6. The Specificity and Sensitivity of LAMP Based on the Fluorescence Signal

Blind samples of *L. monocytogenes* and non-*L. monocytogenes* (N = 100; 40 positive and 60 negative) were amplified by LAMP, and the results were detected as described above. The results from LAMP with colorimetric detection were compared with those of PCR with gel electrophoresis [31]. The specificity of the reaction was defined as the number of true negatives×100 divided by the sum of the number of false positives and true negatives [36]. In parallel, the sensitivity of the reaction (i.e., the detection limit) was determined using 10-fold serial dilutions of cloned *L. monocytogenes* target DNA, as described elsewhere [34,37]. The sensitivity of the LAMP method was examined using 100 blind samples containing 30 real samples, 40 samples spiked with *L. monocytogenes* (500 CFU) and 30 samples without *L. monocytogenes*. The results were compared with those of the PCR assay [31]. The test sensitivity was calculated from the number of true positives×100 divided by the sum of the number of true positives and false negatives [38].

For the spiking test, 10-fold dilutions of bacteria were performed on a salmon matrix ranking from 10^10^ to 0 CFU and incubated as described in a previous section. The bacterial culture was harvested in microcentrifuge tubes, and 50 µL of the bacterial culture dilution was plated onto LM media and incubated at 37 °C for 16–18 h. The number of colonies was counted to determine the number of inocula of the original sample of bacteria.

## 3. Results and Discussion

The successful management of food safety at both the industry and public health levels relies partly on monitoring programs [39].

To date, although several techniques for *L. monocytogenes* detection have been developed, and some are routinely employed [40,41,42], these techniques generally require technical expertise, laboratory facilities, and lengthy periods of time, which are not fit for on-site monitoring or screening purposes. Thus, there is still a need for an affordable assay that is less dependent on laboratory facilities.

To achieve this goal, we combined LAMP with a fluorescence DNA signal detection method and a paper-based lab-on-a-chip platform and developed an affordable and handy assay that does not require any sophisticated laboratory infrastructure in the form of a flexible lab-on-paper chip.

### 3.1. Chip and Chip Fabrication

The paper-based chip design here was based on amplification and detection chemistry, as well as on simplicity and ease of fabrication, and its usage does not require complicated devices, extensive training or large operation costs. To address these demands, a plastic lamination process together with the filter paper, fishing line and plastic materials, all of which are easily accessible and inexpensive, was employed. To make the chip compatible with the DNA assay, all the materials involved were either treated for sterilization (121 °C and 15 psi for 1 h) (paper), or surface sterilized with sodium hypochlorite (plastics) and washed with DNase free water to achieve a nuclease-free environment. The laminate plastic requires an adhesive material and is rinsed with DEPC water to eliminate unwanted nucleases on its surface.

The chip was designed with small dimensions of 2.6 × 4.75 cm^2^ and contains two parts, one for DNA amplification (R for reaction part) and the other for signal detection (D for detection) (Figure 1). DNA amplification occurred in a reaction within Whatman^TM^ filter paper, which served as the amplification pad, at 63 °C for 30 min. Once finished, the reaction solution was squeezed through the connection channel made by the fishing line to facilitate the delivery of a suitable amount of the amplified product (~1 µL) to the volume adjusting pad. Fishing line is better in terms of its flexibility, allowing the fabricated chip to be flexible, unlike the rigid properties of metal wires. In the test, the volume adjusting pad was settled via a small size of filter paper, allowing a fixed volume range of amplified products to be absorbed. In this way, a fixed volume of DNA products could be delivered to the next detection step without using any instruments. Near the volume adjusting pad, there was also a channel at the other arm of the Y, which could be filled with 0.1× phosphate buffer. This was for the DNA dilution to match the amount of DNA binder, which was previously dried using 2 μL of 10^−3^ cyanine dye. Flicking the buffer down through the volume adjusting pad allowed the absorbed DNA (~1 µL) to be dissolved, diluted, and mixed with the DNA binder. After mixing well, the fluorescence signals could be detected after irradiating with UV light if the sample contained amplified DNA from *L. monocytogenes*.

Complete lamination of the chip was performed by heating at 130 °C, which made all the compartments seal, except for the inlet channels for the samples and the buffer solution. For the assay, each chip was pre-filled with LAMP and buffer solution, sealed with a PE sticker, packed in an aluminum foil bag and kept at 20 °C until use. The application of lamination with fishing line for chip fabrication provided several advantages, including its flexible property, easy access, simple manipulation and low cost. Moreover, its flexible property allows the chip to be packed in the appropriated package for outside use with longer storage time. Thus, with just these simple materials, one could fabricate a flexible chip to fit their own purpose. Based on the material cost calculations, a single chip costs less than $0.20 USD. In addition, the fabrication involved simple and easy steps, allowing any scientist with limited experience in device fabrication to independently develop the device based on their needs.

### 3.2. Amplification Platform

DNA amplification via PCR requires two to three different alternating temperature steps. Among them at least one is heating above 90 °C to enable the DNA template a single-strand condition for the enzymatic polymerization. This heating is a significant limitation for the on-chip use. DNA amplification here is based on LAMP. LAMP is an isothermal nucleic acid amplification technique. It reaction takes place at a constant temperature of 60–65 °C using a polymerase with high strand displacement activity [23]. Thus, use of LAMP provides several merits due to its reaction, which does not require a heating step at high temperature to separate the target double-stranded DNA template prior to synthesizing the DNA products [23]. This also helps to avoid bubble problems [43] and provides better and more specific DNA amplification since its reaction employs four specific primers hybridized to six different parts of the target DNA sequence. LAMP also provides several advantages over PCR, such as greater productivity and the use of isothermal temperature conditions, making it fit for the on-chip condition use with only a single incubation step [23,44].

**Figure 2:**
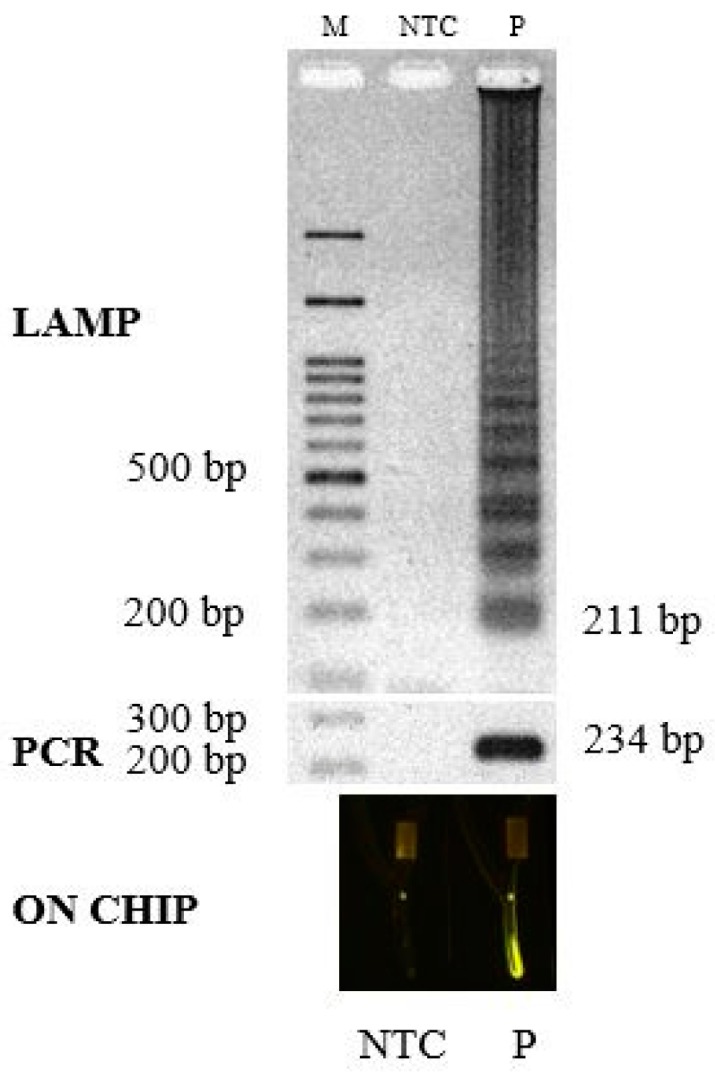
DNA products from the amplification of the *hly* gene by LAMP compared with the PCR products and with detection on a chip; Lane M, 100 base pair ladder; Lane ntc, non-template control; and Lane P, positive DNA from *Listeria monocytogenes*.

In this study, candidate pathogenic determinants of *L. monocytogenes*, including *hly, iap*, *inl*A, *inl*B, *prf*A, and *act*A, were investigated. Among them, the *hly* gene was selected as the target sequence because it is highly conserved among the different strains. The *hly* gene encodes a hemolysin called listeriolysin O [45] that is essential for lysis of the phagosomal membrane. This genetic determinant is also the most commonly used for molecular analysis [31,46,47]. A primer set specific for six areas of the target *hly* gene (from nucleotide position 218 to 428) was obtained (Table 1). Investigation of the specificity of this primer set against *L. monocytogenes* DNA revealed high specificity across all 100 strains of *L. monocytogenes* (based on BLASTn of all primers; reference numbers: SUMGX3XN01R, SUMUN45Z01R, SUSMNH73014, and SUSVYUHW014, 3 January 2014). When *L. monocytogenes* genomic DNA was amplified by LAMP at 63 °C for 40 min, a typical ladder pattern of DNA products with a multiplied size of 211 nucleotides was observed by gel electrophoresis. Positive results of the same size were obtained from *L. monocytogenes-*positive samples. The LAMP results also agreed with those obtained using PCR with the primers in Table 1 [31] (Figure 2).

**Figure 3:**
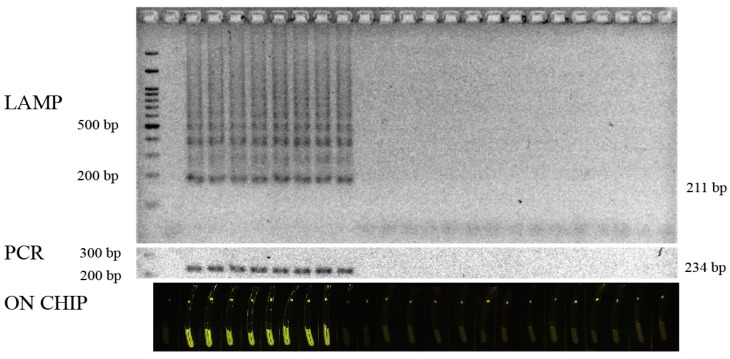
The specificity of the LAMP reaction for *Listeria monocytogenes* compared with that of the PCR and detection on the chip; Lane 1, 100 base pair ladder; Lane 2, non-template control; Lane 3, *L. monocytogenes* plasmid; Lanes 4–10, seven strains of *L. monocytogenes* DNA; Lanes 11–14, four strains of *L. innocua*; Lanes 15–16, two strains of *L. ivanovii*; and Lanes 17–25, DNA from *L. welshimeri*, *Vibrio cholera*, *V. parahaemolyticus*, *Salmonella enteritidis*, *Escherichia coli* O157:H7, *E. coli* ETEC, *E. coli* EPEC, *Pseudomonas putida*, and *Shigella flexneri*.

**Figure 4:**
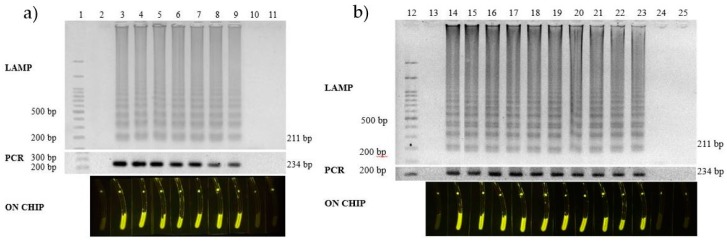
(**a**) The detection limit of the LAMP reaction using DNA as the template compared with that of the PCR and the on-chip assay; Lane 1, 100 base pair ladder; Lane 2, non-template control; Lanes 3–11, 10-fold serial dilutions of *L. monocytogenes* template DNA, ranging from 10^7^ copies to 0 copies. (**b**) The detection limit by spiking the seafood sample with *Listeria monocytogenes* diluents with Lanes 15–19, from 10^10^ CFU to 10^6^ CFU; Lane 20, 1.02 × 10^5^ CFU; Lane 21, 10250 CFU; Lane 22, 1033 CFU; Lane 23, 100 CFU; Lane 24, 7 CFU; and Lane 25, 0 CFU. Lane 12, 100 base pair ladder; Lane 13, non-template control; and Lane 14, *L. monocytogenes* plasmid.

For the specificity assay, DNA from the 22 strains of bacteria, including seven *L. monocytogenes* strains, four *L. innocua* strains; two *L. ivanovii* strains; and one each of *L. welshimeri, Vibrio parahaemolyticus, V. cholera, Salmonella enteritidis, E. coli* O157, ETEC, EPEC, *Pseudomonas putida*, and *Shigella flexneri* (Table 1), were used as templates for LAMP, and the results were compared with that of the PCR [27]. Target gene amplification occurred only in the presence of *L. monocytogenes* (Figure 3). Neither non-specific amplification products nor primer dimers were observed when using non-*L. monocytogenes* strains as templates. Thus, the amplification of the *hly* gene via LAMP undoubtedly occurred only when *L. monocytogenes* was present. Similarly, serial dilutions of a plasmid containing the *hly* gene were employed to determine the sensitivity of the primers. Following amplification, the limit of detection was as low as 100 copies of the *hly* template (Figure 4a). Since the LAMP technique is based on DNA detection, one copy of DNA corresponds to the presence of one bacterial cell in the test system. Thus, the sensitivity in this case was 100 cells. Sensitivity assays based on the spiking of 10-fold serially-diluted bacterial cultures on 50 g of frozen salmon were also carried out, revealing that the limit of detection was 100 CFU in the contaminated frozen seafood, which is similar to the results from the DNA (Figure 4b).

During our study, another report was published on the specific amplification of *hly* using LAMP [45,48]. However, our system targeted amplification at different locations in the *hly* gene and had confirmed primer specificity with *L. innocua*, *L. ivanovi* and *L. welshimeri*, three species in the same genus, whereas the other method did not.

LAMP has been demonstrated on-chip for the detection of pathogens [49]. Most of these studies still focused on the continuous flow of reagents on several chip synthetic substrates [50], all of which required fabrication complexities and professional skills. Our chip is the first report using the application of a simple laminated lab-on-paper chip structure for foodborne detection.

### 3.3. Signal Detection Platform

Fluorescence assays in solution are considered some of the most convenient and sensitive methods. Direct use of fluorescent dyes, which are capable of binding into the grooves of double-stranded DNA (dsDNA) with concomitant fluorescence enhancement, are also matched with the on-chip platform. Fluorescence binders can be cyanine dyes, phenanthridines, or acridine in solution [51]. Here, SYBR Gold^TM^, a simple cyanine DNA binder, was used on the chip. Successful amplification of *hly* allowed this binder to bind the minor groove structure of DNA products, which, in turn, provided subsequent fluorescence signals upon UV illumination.

In the past, our previously developed chip relied heavily on a colorimetric assay via nucleic acid hybridization on blue silver nanoplates. DNA signals could be read by mixing the DNA probe and LAMP products, which performed a DNA hybridization assay to the surface of the nanoparticles; then, adding salt triggered nanoparticle aggregation and a color change [52,53]. A DNA hybridization assay could also be carried out using probe-based fluorescence detection, which involves a sequence-specific fluorescent probe and a fluorescence resonance energy transfer interaction between fluorophores of the donor and of the acceptor during the hybridization of the probe and DNA with increasing and decreasing temperature steps [54,55]. Although the DNA hybridization provided higher fidelity in sequence detection, these aforementioned steps added complexities in arranging the reagents and allocating the steps on the chip structure, thus making it unfit for simple chip application. The use of SYBR Gold^TM^ as a DNA binder here reduced the DNA detection steps to a single one, allowing us to achieve fluorescence signals by the naked eye immediately after DNA amplification with a simple action. Although the assay using DNA binder was less sensitive than nucleic acid hybridization, in theory, its combination with the highly-specific LAMP DNA amplification, organized by six specific priming domains, could compensate for the DNA binder specificity limitation enough for screening and monitoring use. Using this DNA binder also reduced costs compared with the fluorophore probe-based method and provided a chip with a longer shelf life due to dye deterioration when it was used in a dried form.

Fluorescence signals at the bottom of Y were observed in *L. monocytogenes*-positive samples but not in samples lacking *L. monocytogenes*. The specificity and sensitivity of the assay agreed with those obtained by gel electrophoresis and by PCR. However, due to the lower level of DNA template (diluted DNA template less than 100 copies), the test provided less amplification, which produced insufficient DNA products to induce fluorescence signals. Similar results were also observed with PCR.

A sensitivity test was also carried out using live bacteria as the template. The limit of detection result for both the fluorescence signal and gel electrophoresis was also observed when live bacteria were diluted to 100 CFU. It should also be noted that, when non-target DNAs from other LAMP systems (*Escherichia coli*) were employed as templates, the results revealed an induction of fluorescence signals only in the positive *L. monocytogenes* DNA sample (Figure 3). One advantage of LAMP was its DNA productivity, providing a large amount of DNA products even near the limit of detection [37] (Figure 3). This feature finally ensured the success of the fluorescence assay.

Successful signal detection was based on the binding of SYBR Gold^TM^ to the minor groove structure of the target DNA products. When DNA binder was added at the first step and the diluted LAMP products flowed through, the binding of LAMP products with SYBR Gold^TM^ occurred in two different ways. 

In positive LAMP samples, a large amount of several inverted repeats of the target double-stranded DNA *hly* gene produced by LAMP opened up many target minor groove domains for SYBR Gold^TM^, resulting in fluorescence signal induction (excitation and emission for the dye/nucleic acid complex was ~495 nm and ~537 nm, respectively). These signals could be visualized on a chip by the naked eye upon UV exposure (λ = 460 nm) illumination.

In negative LAMP samples, there was no such DNA amplification and no related products available. Then, the addition of SYBR Gold^TM^, providing no fluorescence signal induction, led to no such florescence change. In the test, with 1 µL of LAMP product diluted in 9 μL 0.1× phosphate buffer, a suitable amount of dried SYBR Gold^TM^ was used at 2 μL of 10^−3^ concentration.

In the test, the chip assay was carried out both for a standard test and specificity and sensitivity test. The results revealed a clear fluorescence signal in all the samples, testing positive, and there was almost no signal in all negative samples tested. Additionally, all the results agreed well with those of standard PCR.

### 3.4. Validation of the Test

In total, 100 blind samples were assayed on a chip for the *hly* gene, of which 40 were obtained by randomly spiking known concentrations of *L. monocytogenes* (500 CFU), and the remainder were obtained from a sample without *L. monocytogenes* contamination. These samples were also assayed by PCR for comparison [31]. The spiked concentration of *L. monocytogenes* (500 CFU) was near the limit of detection. Based on these results, the assay using LAMP on the chip and PCR detection, as outlined in Table 3, proved satisfactory. Among the 40 positive samples and 60 negative samples, only one false positive was found, resulting in a standard sensitivity of 100% and a specificity of 98.33%, thus making the assay a good candidate for the screening or monitoring of bacterial contamination in the field [36].

### 3.5. Field Monitoring Application

For field monitoring, frozen and ready-to-eat seafood (N = 30) in the markets were screened using both the PCR assay and the on-site chip. In practical, pieces of seafood (50 g.) were prepared and their surfaces were swapped with a sterile cotton ball. Whole cotton was placed in 15 mL plastic tube previously filled with 1.5 mL of Terrific Broth and incubated at 37 °C in mini water bath, MyBath Model B2000-2 (Benchmark Scientific, NJ, USA) for 3.5 h. One milliliter of bacteria culture was then transferred to a 1.5 mL microcentrifuge tube and centrifuged in a Benchmark MC-12 portable unit, (Benchmark Scientific, USA) at 6000 rpm for 2 min. Pellet of bacteria was collected and 1 μL was suspended in 4 μL of sterile water and all placed into chip inlet No. 1 (previously filled with 5 μL of 2× LAMP solution). After 63 °C incubation in mini water bath, chip was taken out and pressed to deliver the solution to the adjusting pad, mixed with buffer and DNA binder, and checked for its fluorescence signal on UV-LED box, BlueBox Illuminator (Minipcr, MA, USA). Bacterial enrichment at 37 °C and chip incubation at 63 °C could also be carried out in closed polyurethane box filled full with water at a specific temperature in case that no temperature control devices were available, but care should be taken since such box could maintain temperature not more than 30 to 40 min. For a result, only positive samples provide a clear green fluorescence, but not the negative one. The results in Table 4 reveal that one contaminant was found among 30 samples tested (3.33%). Recently, one contaminant was present in the ready-to-eat seafood product.

To date, at least three models for the LAMP-based detection of *L. monocytogenes* have been developed, including those by Tang et al., Wang et al., and Shan et al. [48,55,56]. These studies reported the times required for DNA amplification but not the overall assay time, which includes enrichment. They also relied on non-specific DNA detection via either turbidity changes (due to the aggregation of the magnesium pyrophosphate byproduct in the reaction) [23] or fluorescence based on SYBR green binding to the DNA minor groove. In practice, LAMP could provide a false positive result in the presence of large amounts of interfering DNA or when carryover contamination occurs. In these cases, the test would result in clear turbidity or fluorescence signals that are not due to *L. monocytogenes*. Proper adjustment of the reaction conditions to guarantee reaction specificity is needed.

Here, the use of LAMP increased the DNA signals, and the use of the fluorescence assay provided a similar quality of specificity and sensitivity as the use of SYBR green. This on-chip fluorescence assay also provided enough stability for *L. monocytogenes* with a lower cost and superior ease. These advantages enabled this fluorescence assay to be applied to point-of-care needs.

Thus, through the combination of LAMP and fluorescence signal detection, a simple and rapid lab-on-a-chip assay applicable to the on-site monitoring of *L. monocytogenes* was developed. In addition, the test platform is a ready-to-use on-site application because LAMP can be easily tailored for different uses and because SYBR Gold^TM^ is stable after drying during six months of storage. This approach shows considerable merit, both for its ability to rapidly yield satisfactory results and for lessening the dependence on laboratory facilities for field monitoring testing. Although this assay is qualitative, the total cost was as low as approximately $5 USD per test ($0.2 USD for the chip, $4 USD for the LAMP enzyme, reagents and DNA binder, $0.3 USD for the package, and $0.5 USD for the bacterial culture) compared with $15 USD for those of PCR assay or $75 for real-time PCR or $10 USD for immuno capture ELISA (Thailand rate). Even when it was compared with immuno chromatographic (lateral flow) test whose cost were around $4–$5 USD with almost one day retention time requirement, our chip provided more time merit with the entire procedure required less than 4.5 h to complete (3.5 h for enrichment and 30 min for the *L. monocytogenes* assay). The cost of the test could be lower if it is produced en mass. Since the chip here was designed for test monitoring at industrial and authority level, all those advantages could provide an alternative for monitoring *L. monocytogenes* in the field, especially for food quality and safety purposes.

## 4. Conclusions

A rapid lab-on-paper chip assay for the detection of *L. monocytogenes* (*hly* gene) was developed. The assay was based on DNA amplification by LAMP and the detection of DNA products by a fluorescence assay all in a single chip platform. Although the on-chip assay here provided less specificity (98.33% compared with 100% of PCR), the test provided equal sensitivity (100%), with a detection limit of 100 copies of the target *hly* gene, better than that of immunological methods which have specificity and sensitivity somewhere between 70% to 85% [56]. Additionally, the total cost was less than $5 USD per test, and the overall process took less than 4.5 h to complete, including both bacterial enrichment and DNA analysis. On-chip assay required only mini water bath, small centrifuge device, UV LED box, and autopipette, suitable for field applications. Applications of the lab-on-paper chip for screening assays revealed one contamination out of 30 samples from a low sanitation vendor in local markets, which all agreed with the PCR results.

## Figures and Tables

**Table 1:** Corresponding primers and probes used for amplification of *L. monocytogenes*.

Name	Sequence (5′ to 3′)	Reference Position *
F3	ACAATGTATTAGTATACCACGGA	218-240
B3	TCTGGTTGATTTTCTACTAATTCC	405-428
FIP	GGATTTCTCTTTTTCTCCACAACGTTTTGATGCAGTGACAAATGTGC	(297-321)–(241-259)
BIP	GCAGACATCCAAGTTGTAAATGCTTTTCGCTTTTACGAGAGCACC	(337-359)–(382-399)
PCR Forward Primer **	CGGAGGTTCCGCAAAAGATG	1044-1036
PCR Reverse Primer **	CCTCCAGAGTGATCGATGTT	1258-1277

* With reference to sequence accession number AF253320.1 (GenBank); ** [31].

**Table 2:** Bacterial strains used in this study and their sources.

No.	Bacteria	Strain
1	*Listeria monocytogenes*	DMST * 17303
2	*L. monocytogenes*	DMST * 1327
3	*L. monocytogenes*	DMST * 20093
4	*L. monocytogenes*	DMST * 23145
5	*L. monocytogenes*	DMST * 31802
6	*L. monocytogenes*	FRTL ** 1299
7	*L. monocytogenes*	FRTL ** 1401
8	*L. innocua*	DMST * 9011
9	*L. innocua*	FRTL ** 1265
10	*L. innocua*	FRTL ** 1445
11	*L. innocua*	FRTL ** 1446
12	*L. ivanovii*	FRTL ** 1243
13	*L. ivanovii*	DMST * 9012
14	*L. welshimeri*	DMST * 20559
15	*Vibrio cholera*	FRTL ** 1322
16	*V. parahaemolyticus*	FRTL ** 0886
17	*Salmonella enteritidis*	DMST * 15676
18	*Escherichia coli* O157:H7	DMST * 12743
19	*E. coli* (ETEC)	DMST * 30543
20	*E. coli* (EPEC)	DMST * 30546
21	*Pseudomonas putida*	DMST * 16074
22	*Shigella flexneri*	DMST * 4423

* DMST = Department of Medical Sciences, Ministry of Public Health Thailand; ** FRTL = Food Research and Testing Laboratory, Faculty of Science, Chulalongkorn University, Thailand.

**Table 3:** Comparison of PCR with LAMP on-chip detection.

Specimens	PCR	LAMP on Chip
Positive	40	41
Negative	60	59
False positive	-	1
False negative	-	0
Sensitivity ^A^		100.00
Specificity ^B^		98.33

^A^: Sensitivity = 100 × true positive/(true positive + false negative); ^B^: Specificity = 100 × true negative/(false positive + true negative).

**Table 4:** On-site screening of products from modern supermarkets and locals.

Number	Sample	Collecting Location	Result	
			on PCR	on Chip
1	Salmon filet portion sashimi	Supermarket	N	N
2	Raw salmon meat (block)	Supermarket	N	N
3	Raw salmon meat	Supermarket	N	N
4	Smoked salmon	Supermarket	N	N
5	Salmon	Supermarket	N	N
6	Fresh cut salmon	Supermarket	N	N
7	Frozen peeled shrimp	Supermarket	N	N
8	Fresh shrimp sashimi	Supermarket	N	N
9	Raw tuna sashimi	Supermarket	N	N
10	Raw tuna block for sashimi	Supermarket	N	N
11	Raw salmon meat for sashimi	Supermarket	N	N
12	Raw salmon halve dressing	Supermarket	N	N
13	Fresh cut salmon (disc)	Supermarket	N	N
14	Frozen salmon sashimi	Supermarket	N	N
15	Repacked smoked salmon	Supermarket	N	N
16	Raw salmon for sashimi	Local market	N	N
17	Raw salmon sushi	Local market	N	N
18	Fresh shrimp	Local market	N	N
19	Frozen shrimp peeled clean tail on	Local market	N	N
20	Uncooked shrimp easy peeled tail on	Local market	N	N
21	Ready-to-eat salmon sushi	Local market	P	P
22	Repacked smoked salmon	Local market	N	N
23	Raw salmon for sushi	Local market	N	N
24	Raw salmon for sushi	Local market	N	N
25	Frozen salmon block	Local market	N	N
26	Raw shrimp peeled tail on	Local market	N	N
27	Raw salmon block	Local market	N	N
28	Fresh shrimp for raw serve	Local market	N	N
29	Fresh shrimp for raw serve	Local market	N	N
30	Salmon for sushi	Local market	N	N

N = Negative; P = Positive.
